# From Food Waste to Functional Biopolymers: Characterization of Chitin and Chitosan Produced from Prepupae of Black Soldier Fly Reared with Different Food Waste-Based Diets

**DOI:** 10.3390/foods13020278

**Published:** 2024-01-16

**Authors:** Alessia Mannucci, Luca Panariello, Linda Abenaim, Maria Beatrice Coltelli, Annamaria Ranieri, Barbara Conti, Marco Santin, Antonella Castagna

**Affiliations:** 1 Department of Agriculture, Food and Environment, University of Pisa, Via del Borghetto 80, 56124 Pisa, Italy; alessia.mannucci@agr.unipi.it (A.M.); linda.abenaim@phd.unipi.it (L.A.); anna.maria.ranieri@unipi.it (A.R.); barbara.conti@unipi.it (B.C.); antonella.castagna@unipi.it (A.C.); 2 Department of Civil and Industrial Engineering, University of Pisa, Via Diotisalvi 2, 56122 Pisa, Italy; luca.panariello@ing.unipi.it (L.P.); maria.beatrice.coltelli@unipi.it (M.B.C.)

**Keywords:** *Hermetia illucens*, sustainability, chitin, chitosan, food waste, acetylation degree

## Abstract

The use of food waste as a rearing substrate to grow insects is an ecofriendly and sustainable alternative to food waste disposal. In the present research, *Hermetia illucens* prepupae were reared with a standard diet, different food waste-based diets based on vegetables, fruits, and meat, and a mixed one, where the previous three components were present equally. The demineralization and deproteination of the prepupae allowed for the obtainment of chitin that was then deacetylated to produce chitosan. Also, the bleaching of chitosan was attempted for further purification. The yield of the different reactions was investigated, and the infrared spectra of the obtained materials were analyzed to obtain information on the quantity and acetylation degree trend of the chitin and chitosan as a function of the diet. The possibility to slightly modulate the yield and acetylation degree of both biopolymers thanks to the specific diet was enlightened. Interestingly, the standard diet resulted in the highest fraction of chitin having the highest acetylation degree, and in the highest fraction of chitosan having the lowest acetylation degree.

## 1. Introduction

Reducing food loss and waste is one of the main challenges worldwide to achieve the goals of ensuring food access to as many people as possible, reducing the ecological footprint of the food production chain, and providing economic and social benefits. According to the UNEP Food Waste Report 2021, though data on food waste are fragmented and there are little available, about 17% of total global food production may be wasted. Apart from the measures that must be adopted at the different food chain levels to prevent food loss and waste, the reframing of waste management and selection of options other than material disposal is absolutely needed. Among the different possibilities, the use of food wastes to rear bio-converting insects represents a valuable method to recycle such wastes. In fact, the current trend is to use insects reared on food waste for the production of many valuable substances such as biofuels, fertilizers, pharmaceuticals, proteins, lipids, and animal feed in a circular economy perspective [1].

*Hermetia illucens* (Linnaeus, 1758) (Diptera Stratiomyidae), also known as Black Soldier Fly (BSF), is an ecologically valuable insect whose usefulness in organic waste recycling and animal feed production has attracted increasing attention in recent decades. It is, moreover, a promising source of chitin to produce chitosan [2,3,4]. Though BSF exuviae have the highest chitin content [5], the prepupal stage is easier to collect at the laboratory level.

Chitin (β-(1-4)-N-acetyl-D-glycosamine) is one of the most abundant natural polysaccharides as it is a constituent of Arthropod exoskeletons and fungal cell walls. Among Arthropods, shrimps and crabs are characterized by a high chitin content (20–30%) and are the most common commercial sources of this polysaccharide [6]. However, as the global demand for chitosan is on the rise, alternative sources will have to be found in order to meet market demand. Indeed, research has also focused on the study of insects, characterized by 10–15% chitin, as an alternative source of the latter [7,8,9,10,11]. The extraction of chitin from insects is considered advantageous because of the ease of rearing, the very short production time, and the possibility of using organic waste materials to feed the insects [11]. The most investigated insect species for chitosan production are *Tenebrio molitor* (Linnaeus, 1758) (Coleoptera Tenebrionidae), *Alphitobius diaperinus* (Panzer, 1797) (Coleoptera Tenebrionidae), and, in particular, *Hermetia illucens* (Linnaeus, 1758) (Diptera Stratiomyidae). Chitosan has well-known antimicrobial, antioxidant, and antitumoral properties, and its application has spread to many sectors, including the pharmaceutical, medical, veterinary, food [12,13], and agricultural sectors. In agriculture, in particular, chitosan is applied to soil as a biostimulant for plant growth and abiotic stress tolerance [14,15], promoting symbiotic interaction between plants and microorganisms, and improving the metabolisms of fruit, plants, and germination against insect pests [16]. Chitosan can be chemically derived from chitin through a partial deacetylation process that removes around 80% of the acetyl groups and allows the obtaining of this polysaccharide, which is well-known for its film-forming and antimicrobial/antioxidant properties [17,18]. Indeed, chitosan can be easily solubilized in a dilute acidic medium, resulting in a solution that can be used as ecofriendly food packaging, as film, or as edible coatings [18,19,20]. The production of chitosan involves three main steps: demineralization, deproteinization, and final deacetylation [21]. The first one needs the use of an acid solution to remove minerals, mainly calcium carbonate [3], while the other steps require strong bases to remove proteins and part of the acetyl groups. Altering the time, temperature, and solution concentration might lead to chitosan with a different acetylation degree, average molecular mass, and dispersity, which affects chitosan solubility and other properties [14]. Mild acidic conditions, for instance, lead to the production of chitin nanofibrils, consisting of nanometric solid fibrils, representing the crystallin part of chitin, and showing an acetylation degree similar to it. However, fibrils have a low acetylation degree on their surface. Due to their surficial properties and enhanced surface-to-volume ratio, they show anti-microbial properties like chitosan [22]. They form stable suspensions in slightly acidic water solutions. Moreover, their application, as the one of chitosan, enhanced barrier properties of biopolymer substrates [23], suggesting applications in the sector of bionanocomposites for the packaging [22,24] and personal care sector [25,26]. The control of the deacetylation reaction and the final acetylation degree is, thus, pivotal for modulating the macromolecular structure and morphology of the final product.

The disposal of food waste is economically and environmentally costly, so, in this research, we exploited the ability of *H. illucens* to bioconvert such wastes into precious compounds such as chitin and chitosan. Despite the publication of recent studies on chitin extraction and chitosan production from insects [8,9] or, specifically, from *H. illucens* [3,4], there are scarce information about the effect of different diets on chitin and chitosan yield extracted from *H. illucens*. Only Eggink et al. [27] have investigated the hypothetical influence of rearing substrate on larval chitin content. The study found that larvae reared on poultry feed or mixed feed had a higher chitin content, even if the chitin content seemed to be more influenced by larval development.

For this reason, this paper aims to investigate the bioconversion of different organic wastes for chitin and chitosan production by BSF. The primary objective of the work was to understand whether, and to what extent, different organic waste diets could influence the yield and physico–chemical characteristics of chitin and chitosan extracted from BSF prepupae.

## 2. Materials and Methods

### 2.1. Hermetia illucens Rearing

*Hermetia illucens*, throughout the whole cycle, has been reared under laboratory conditions (T. 30 °C, R.H. of 60%, 16 h light/8 h dark photoperiod, and irradiance of 1500 lux) at the Department of Agriculture, Food, and Environment of the University of Pisa (Italy) since November 2019. The rearing consists of adult cages, oviposition supports for egg collection, and boxes for the development of pre-imaginal instars. The adult cages (47.5 × 47.5 × 93 cm) are of the model BugDorm-4M4590DH, manufactured by Mega View Science Co., Ltd. in Taichung, Taiwan. These cages, made of polyester, have knitted mesh for good air circulation and are illuminated by a 1500 lux LED panel light. Inside each adult cage, water and sugar containers are always available, and a plant branch is provided as a resting place for the adult and a place to perform their leaking behavior [28]. As females are attracted to decomposing organic material for egg laying, wooden oviposition supports (20 × 3 × 1 cm) are placed inside the cage on top of plastic boxes filled with rotting fruit (usually apples in decomposition). The eggs are removed from the supports after 2 days and transferred to other plastic boxes (29 × 18 × 9 cm) containing artificial food (poultry feed and water) for future larvae. When the pupal stage is reached, the plastic boxes are transferred to a new adult cage to facilitate the emergence of the next adult generation.

### 2.2. Hermetia illucens Prepupae Production on Different Diets

For the experiment, *H. illucens* larvae were fed on five different diets (standard, fruit, vegetable, meat, and mixed diet) as reported in Table 1.

Diets were ground in a Moulinex Perfectmix grinder, operating at 800 W for 4 min. Then, 1000 g of each diet were put in boxes (29 × 18 × 9 cm). About 1000 six-day-old larvae were placed inside the boxes containing the specific diets, and three replications were performed for each diet. Once the pupal stage was reached, the prepupae were washed with distilled water, dried over an absorbent paper, and stored at −20 °C before the chitosan extraction process.

### 2.3. Evaluation of Hermetia illucens Larval Mortality

To confirm the suitability of the different diets used, larval mortality was assessed. The latter was evaluated on 30 larvae reared separately for each diet (standard, fruit, vegetable, meat, and mixed). The experiment has been replicated five times for a total of 150 larvae/each diet.

The larvae were maintained in PE cups (5 cm diameter × 8 cm height) with 30 g for each diet until all larvae reached the pupal stage. Mortality was calculated using the following formula:

BSF larval mortality (%) = [(total number of larvae − number of survival)/total number of larvae] × 100 (1)

### 2.4. Chitin Extraction and Chitosan Production from Hermetia illucens Prepupae

The extraction of chitin and the production of chitosan from prepupae samples (3 biological replicates) reared with the different diets was performed following the process reported by Hahn et al. [21] with some modifications. First, the raw material was dried in an oven at 50 °C and ground into a fine powder before starting the extraction process. The samples were treated with 0.5 M formic acid (1:10, *w*/*v*), stirred for 30 min at room temperature to remove minerals, and then filtered through non-woven tissues. We rinsed them multiple times with distilled water until they reached a neutral pH. After that, we dried the neutralized samples in the oven at 50 °C until completely dry.

To get rid of proteins, the samples were treated with 2 M sodium hydroxide and stirred for 2 h at 80 °C. The deproteinized samples (chitin) underwent the same process as the demineralization step to neutralize them and were finally oven-dried.

For making chitosan, chitin samples were then deacetylated using 12 M sodium hydroxide (60 g DW: 800 mL) and stirred continuously for 3 h at 90 °C. Afterward, the samples were washed as before and dried in the oven.

A bleaching step was added by soaking them in a 5% (*v*/*v*) hydrogen peroxide solution (1:20, *w*/*v*) for 15 min at 90 °C. Afterwards, bleached chitosan samples were neutralized and dried as previously described. The bleaching step in the process was undertaken with the specific aim of eliminating any natural pigmentation in the chitosan samples, ensuring that the chitosan obtained is more visually neutral and suitable for various downstream applications.

The chitin and chitosan yield (%) was calculated for all the samples as the ratio between chitin or chitosan (g DW) and the starting raw prepupae material (g DW). The rate between demineralized and raw dried starting biomass, bleached chitosan/chitosan, and chitosan/chitin rate were also reported as a percentage of dry weight (DW).

### 2.5. Chitin and Chitosan Characterization

#### 2.5.1. ATR-IR Characterization

Chitin and chitosan powders were crushed and homogenized using a pestle in a mortar. To perform infrared characterization, powder was transferred from the mortar to the ATR crystal using a spatula. Infrared spectra were recorded in the 550–4000 cm^−1^ range with a Nicolet 380 Thermo Corporation Fourier Transform Infrared (FTIR) Spectrometer (Thermo Fisher Scientific, Waltham, MA, USA) equipped with a smart Itx ATR (Attenuated Total Reflection) accessory with a diamond plate, collecting 256 scans at 2 cm^−1^ resolutions. At least three replicates from each batch were analyzed. For each sample, the RAC ratio was calculated and correlated to the acetylation degree of the sample, and it was determined as reported by the following equation:

R_AC_ = A_amide_/A_reference_ (2)

where A_amide_ is the area of the band obtained by integrating the peak at 1650 cm^−1^ related to the CO stretching (Amide I) in the range of 1695–1618 cm^−1^, and A_reference_ is the area of the reference band in the range of 1184–1024 cm^−1^ corresponding to C-O-C stretching movements. The integrals were measured after tracing a baseline passing through the minima present in all the spectra at about 1735 cm^−1^ and 1185 cm^−1^. EZ OMNIC software (OMNIC 7.2, Thermo, Waltham, MA, USA) was used to elaborate the spectra and measure the integrals.

#### 2.5.2. SEM Analysis

The morphology of chitin powders was investigated with an EM 30 scanning electron microscope (SEM) (Coxem Ltd., Daejeon, Republic of Korea). Powders were mounted on stub and sputtered with gold on with a sputter coater Edward S150B (Edwards High Vacuum International, Crawley, UK) to avoid charge build-up and grant an intense secondary electron signal.

### 2.6. Statistical Analysis

The percentage of total larval mortality of *H. illucens* larvae was reported as the average of the percent larval mortality of the different replicas. The Shapiro–Wilk normality test (JMP software, JMP^®^, Version 16. SAS Institute Inc., Cary, NC, USA, 1989–2021) indicated an abnormal distribution of data. Therefore, the nonparametric Kruskal–Wallis Test (*p* < 0.05) was employed.

Differences in the yield and acetylation degree among the different experimental groups were assessed through one-way ANOVA followed by post hoc Tukey–Kramer test (*p* < 0.05, JMP software, JMP^®^, Version 16. SAS Institute Inc., Cary, NC, USA, 1989–2021). All measurements were performed in triplicate, and data are presented as the mean ± standard error. ATR-IR and SEM analysis were performed in triplicate for each sample to confirm similarity and one of each sample was reported.

## 3. Results and Discussion

### 3.1. Effect of Diets on H. illucens Larval Mortality

**Figure 1:**
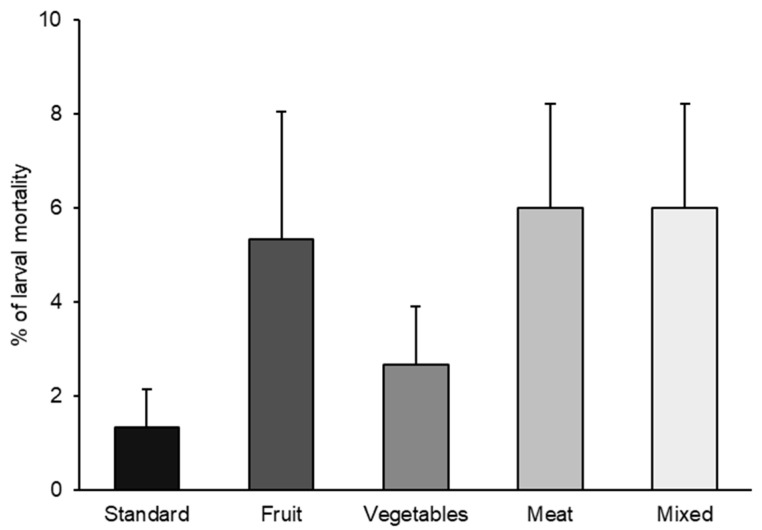
BSF larval mortality (%) reared with the five different diets (standard, fruit, vegetable, meat, and mixed). Data represents the mean ± SE (*n* = 5). For each column significance level at *p* < 0.05 (X^2^ = 7.75; DF = 4; *p* = 0.1008).

As *H. illucens* is currently produced for animal feed on many European farms, and in the EU, the waste allowed for its feeding is mainly limited to agro-industrial vegetable waste (Regulation (EC) No 767/2009, Annex III and Regulation (EC) No 68/2013 Directive 2008/98/EC) [29], it is important to assess the suitability of these substrates for larval rearing. Indeed, our results did not show a statistically significant effect of diets on larval mortality (*p* = 0.1008) (Figure 1). 

The highest larval mortality percentage (6.0%) was observed for substrates prohibited in the EU, namely meat and mixed diets. The next highest percentages were noted for fruit and vegetable diets (5.3 and 2.6%, respectively), which are EU-allowed substrates. The lowest mortality was recorded for the standard diet (1.33 ± 0.81%).

In the present study, larval mortality was very low for all diets (less than 6%), and these results agree with other research in which BSF larvae were reared on different organic waste-based diets and by-products, especially based on vegetables and fruits [30,31,32]. Our results indicated that all used substrates can effectively support the larval development of the species.

### 3.2. Influence of the Diet on Biomass Recovery

**Figure 2:**
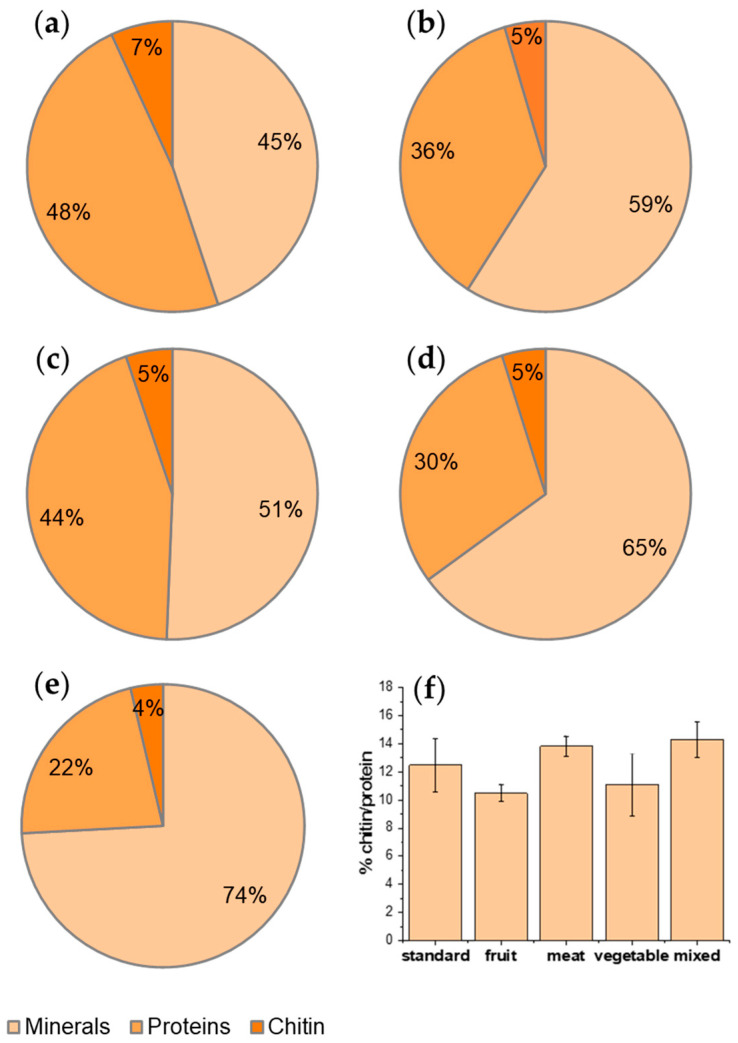
Percentage composition in terms of minerals, proteins, and chitin with respect to raw material from BSF reared with (**a**) standard diet; (**b**) vegetable diet, (**c**) fruit diet; (**d**) meat diet; (**e**) mixed diet. (**f**) Percentage ratio of chitin with respect to protein fraction.

Table 2 shows the biomass recovery for each step of the process. Looking at the impact of the processes in terms of recovery, it is evident that demineralization and deproteinization steps accounted for a biomass loss total accounting for about 95% of the starting material. Hahn et al. [4] reported a low content of minerals (mainly calcium and magnesium salts) and a high protein content in BSF that, according to the literature, should be around 38% in pupal exuviae and 49% in adults of BSF [3]. In our case, the estimation of protein and mineral percentage composition of BSF biomass indicates a predominant mineral percentage, apart from the standard diet (Figure 2). The mineral content is higher for a meat-based diet and for a mixed diet, suggesting that these diets favor the fixation of minerals in BSF exoskeletons, where minerals are mainly present. But, if the rearing, as usual, is aimed at producing proteins and chitin from waste, these two diets are less convenient than the others and the best diet is the standard one followed by the one based on fruits and the one based on vegetables.

Interestingly, observing the recovery trend, the highest content of protein and chitin, with respect to the starting raw material, was obtained by feeding *H. illucens* with the standard diet, mainly based on cereals with a high content of cellulose and starch, whereas the mixed diet resulted in the highest mineral content (Figure 2a–e). The chitin content, with respect to the raw material, has the highest value for the standard diet. Nevertheless, if the percentage ratio of chitin, with respect to the protein fraction, is calculated (thus excluding the mineral fraction, not the organic fraction, that represents waste), 14.3% is obtained for the mixed diet and 12.5% for the standard diet, meaning there is no significant difference (Figure 2f). This ratio is slightly lower only for fruit with respect to meat and mixed feeding, where the protein (and, thus, the nitrogen, which is also present in the chitin macromolecules and, thus, necessary for its biosynthesis) content of the feeding is higher. These results suggest that a diet richer in protein can allow for the ratio of chitin/protein to be slightly higher.

**Figure 3:**
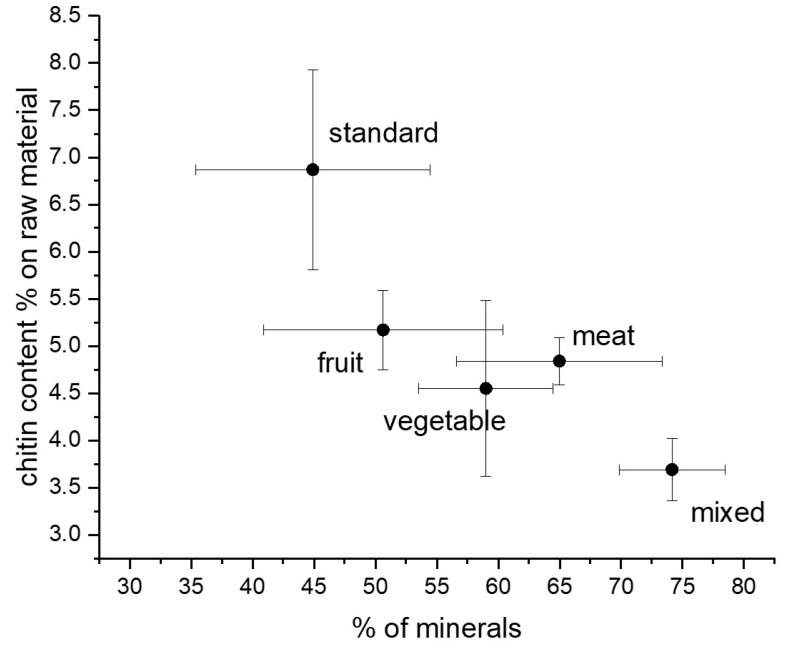
Chitin content versus the percentage of minerals in the *H. illucens* prepupae.

Interestingly, a decreasing trend of chitin content was found as a function of the mineral content of the prepupae (Figure 3). This result is surprising because chitin is mainly present in the exoskeleton of the insect, which contains minerals. Hence, the opposite trend could have been expected [33]. As shown in Figure 2f, on the contrary, an almost constant ratio with respect to protein content was found in *H. illucens* prepupae. These results can be explained considering the strong connection between chitin and protein layers in insect cuticles [34]. Chitin is, in fact, embedded into a protein matrix. And differences in the protein profile and in the chitin/protein ratio, together with the presence of minerals, account for the specific properties of the cuticle according to the anatomical location and the insect developmental stage [35]. On the whole, a standard diet based on cereals or diets based on fruits and vegetables are more convenient for rearing BSF because of the higher recovery in both protein and chitin with respect to the raw dried starting biomass. These considerations are furthermore illustrated in Figure 3, reporting the chitin content of samples as a function of their mineral content.

A research paper conducted on BSF larvae reared on different substrates reports that the content of chitin (determined by chromatographic quantification of glucosamine produced by the acidic hydrolysis of chitin) increased as a function of larvae age but also showed variation attributable to the rearing substrate [36]. Differently, in our study, no significant effect due to the diet factor was observed on the yield after the demineralization and deproteinization steps (Table 2). Moreover, the chitosan yield, irrespective of the bleaching step, was not affected by the different rearing substrates. This is in line with what was found by Meneguz et al. [37], who did not notice any significant differences in the chitin content of BSF larvae reared on a vegetable and fruits mixture or a mixture of fruits, although some differences were evident for the crude protein, ash, ether extract, and detergent fibers. However, in their study, chitosan production was not investigated.

The rate of unbleached chitosan/chitin showed no significant differences (Table 2). For this parameter, our recovery rate was higher than the one obtained by Triunfo et al. [3], which ranged between 28–42% for pupal exuviae and adults. Bleached chitosan showed a particularly low biomass recovery rates, which were 14–19% in respect to unbleached chitosan (Table 2), but also this parameter was unaffected by diet factor.

Unfortunately, the scientific literature considering chitin and chitosan from BSF prepupae is scanty and for this reason we were able to compare our data with very few studies, which, however, differed in terms of BSF growth stage and methods applied for the extraction. Indeed, the variety of methods, chemicals, time, and temperatures that can be used for chitin extraction and chitosan production will probably affect the yield even starting from the same source, so it should be considered that changes in some of these factors might lead to different yield and quality results.

### 3.3. ATR-IR Analysis

**Figure 4:**
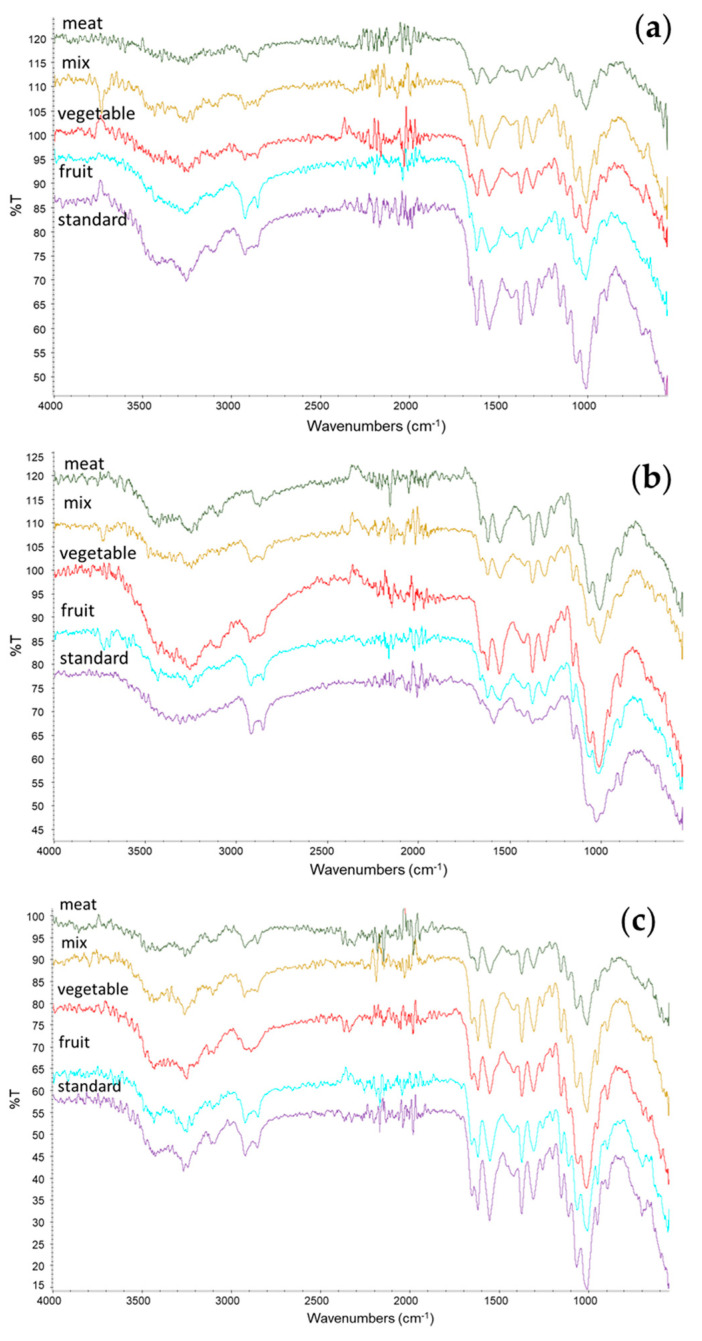
ATR-IR spectra of (**a**) chitin, (**b**) chitosan, and (**c**) bleached chitosan from *H. illucens* prepupae reared with different diets.

The chemical structures of chitin and chitosan were investigated using ATR-IR analysis. The resulting spectra of chitin, chitosan, and bleached chitosan were reported in Figure 4a, Figure 4b, and Figure 4c, respectively. Characteristic peaks of chitin can be identified in all samples at 1310–1320 cm^−1^ (CN-stretching, Amide III), 1550–1560 cm^−1^ (NH-bending, Amide II), and 1650–1655 cm^−1^ (CO-stretching, Amide I). The Amide I band splits, around 1620 and 1650 cm^−1^, were reported as typical of the α-form of chitin [3]. The deacetylation process that led to chitosan can be identified by the intensity reduction of the amidic band into the amine band and, in particular, the band at 1590 cm^−1^ (NH_2_ bending). The increment of this band can be observed in all the IR spectra of chitosan and, in particular, in the standard sample, where the band is clearly visible (in the other samples, this was overlayed by the Amide II band). No significant differences were observed between the spectra of bleached chitosmidean and the respective unbleached samples.

**Figure 5:**
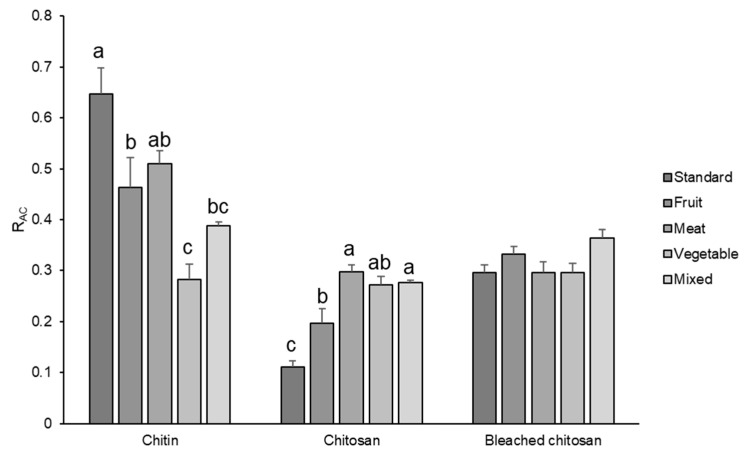
R_AC_ of chitin, chitosan, and bleached chitosan from *H. illucens* prepupae reared with different diets. Data represent the mean ± SE (n = 3). Different letters indicate statistically significant differences according to one-way ANOVA followed by Tukey–Kramer post hoc test (*p* < 0.05).

R_AC_ values of chitin, chitosan, and bleached chitosan samples were calculated for each set of spectra and reported in Figure 5. These results indicate that diet influenced the acetylation degree, which displayed the highest values for the standard diet and the lowest values for the vegetable one. In particular, chitin extracted from insects grown with fruit, vegetable, and mixed diets showed significantly lower R_AC_ values in respect to the standard substrate (−28.3, −56.3, and −40%, respectively).

The partial deacetylation of chitin in the insect cuticle, conducted by chitin deacetylase (CDA), modifies some characteristics of the cuticle, among which are the resistance to endochitinase-mediated hydrolysis, the protein-binding properties, and even the affinity for specific proteins [35]. According to our results, CDA activity seems to be influenced by the rearing substrate. Indeed, the concentration of the acetyl group on the chitin macromolecules in *H. illucens* prepupae depends significantly on the diet. The elevated level of acetylation (>50%) in chitin delimits its use in applications requiring chitin to exist in nanofibril form due to its poor solubility. Therefore, if the rearing of this insect is aimed at obtaining chitin nanofibrils [6,24,25,26], the vegetable diet seems to be the worst solution because it results in the chitin with the lowest acetylation degree. Thus, it is the one that can be better solubilized in an acid solution.

Integrating the results of Figure 5 with the one obtained by the mass recovery in the previous section, it is possible to deduct that a standard diet can result in the highest content of chitin with the highest acetylation degree. Fruit-, vegetable-, and meat-based diets resulted in a similar content of chitin (Figure 3), but the acetylation degree was significantly lower for the insects fed with a vegetable-based diet. Moreover, considering the similar acetylation degree, a fruit-based diet can be convenient in rearing BSF with respect to a meat-based diet, because for the latter, a lower content of proteins is obtained.

**Figure 6:**
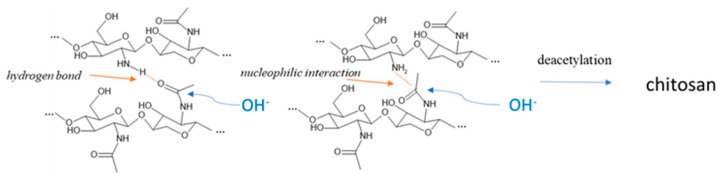
Inter-macromolecular interactions between glucosamine and N-acetyl glucosamine units limiting the deacetylation reaction due to the sodium hydroxide.

R_AC_ of chitosan (Figure 5) revealed a completely different trend. The lowest value was indeed observed in chitosan obtained from prepupae reared on the standard diet, according to the IR results where the band relative to the amine group was very intense. All the other samples showed higher R_AC_ values, with respect to the standard diet (+77.5% for fruit, +169.5% for meat, +146.0% for vegetables, and +149.6% for mix), but they were still lower compared to the chitin, except for the vegetables-based diet, which remained unchanged. The decrease in R_AC_ due to deacetylation is the highest for the standard diet and the lowest for the vegetable diet. These results evidenced a more efficient deacetylation process on the standard sample that presented the higher availability (and density) of the amidic group on the carbohydrate chain. Considering Deringer et al.’s work [38], it is possible to consider that, in α-chitin, the occurrence of hydrogen bonding is present between the chitin macromolecules arranged in an anti-parallel way. In the case of a more deacetylated chitin, like the one obtained from prepupae fed with a vegetable diet, the occurrence of hydrogen bond and nucleophilic interactions is enhanced, especially in the amorphous more disordered fraction of chitin, due to the higher nucleophilic character of the nitrogen in amine groups with respect to nitrogen in N-acetyl groups. Thus, the attack of hydroxide anions during the deacetylation is discouraged with respect to a chitin with a higher acetylation degree (Figure 6).

The bleaching process (Figure 5) further changed the R_AC_ values bringing all samples to a mean value of 0.3, with no significant differences among samples. This phenomenon could be attributed to the acidification step of the bleaching process where the low acetylated molecules were very soluble and were lost during the washing step. Hence, the bleaching, which resulted in a strong mass loss in chitosan, led to the elimination of the most reactive and soluble fraction of this polymer.

### 3.4. SEM Analysis

**Figure 7:**
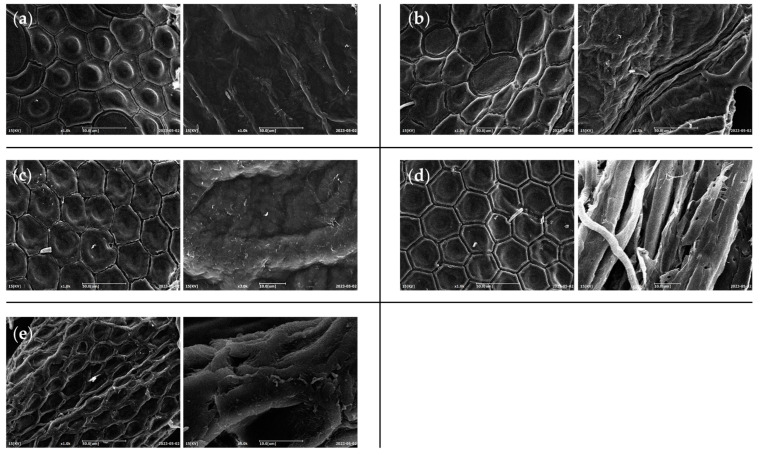
Micrographs of chitin powders of *H. illucens* reared with (**a**) standard, (**b**) fruit, (**c**) meat, (**d**) vegetable, or (**e**) mixed diet, obtained by SEM analysis.

The morphology of different chitins was investigated with SEM in order to evidence any differences in the typical surface morphology of the samples obtained by *H. illucens* that might be due to the influence of different diets. Representative micrographs were reported in Figure 7.

All the micrographs exhibited the typical honeycomb-like structure mainly composed of a repetition of hexagonal units [3]. All chitins showed a surface structural complexity similar among them, with the slight tendency of standard and meat diet fed prepupae in showing a more reduced distance between hexagon borders with respect to the other samples. Triunfo et al. [3] showed how the chitin surface could vary among different stages, where adults of *H. illucens* displayed a higher complexity when compared to larval and pupal exuviae. From our SEM images, we can conclude that diet is a factor without a great influence at this level.

## 4. Conclusions

This is the first study investigating the influence of different diets, based on food-waste substrates, on the quality and quantity of chitin and chitosan derived from *H. illucens* prepupae. All the diets tested showed low larval mortality without any significant difference among the diets, demonstrating the suitability for *H. illucens* rearing all of them. As expected, the biomass recovery produced from prepupae reared with these different waste-based substrates did not show any significant differences as well. The percentage of minerals and proteins depends strongly on the diet, with the standard diet resulting in the lowest mineral content, whereas the ratio between chitin and protein is less affected by the diet. Regarding the chitin characterization, the acetylation degree varied according to the different diets used in this study, indicating the possibility of modulating this feature, with the vegetable diet leading to a less acetylated material that maintained similar values after the deacetylation process. After chitosan production, an influence of the diet factor was still evident, with the standard diet showing the lowest acetylation degree, probably due to a more effective process linked to the higher availability of the amidic group on the carbohydrate chain. Though the bleaching step resulted in a substantial loss of chitosan mass and of the more soluble and reactive component of the chitosan, this process caused a R_AC_ leveling, and this should be considered as a chance to obtain a similar acetylation degree, and then a likely similar final product from *H. illucens* reared on different substrates. By efficiently recycling different food wastes, ultimately leading to the production of multifunctional chitin and chitosan polymers, this approach fits well with the principles of circular economy.

## Figures and Tables

**Table 1:** Composition of the five diets used for the experiment.

Diet	Composition
Standard	Poultry feed and water (40:60)
Fruit	75% apple, orange, banana (1:1:1) + 25% standard diet
Vegetable	75% celery, sweet pepper, potatoes (1:1:1) + 25% standard diet
Meat	75% poultry meat + 25% standard diet
Mixed	25% for each previous diet (fruit, vegetable, meat and standard) (1:1:1:1)

**Table 2:** Biomass recovery (%) after chitin extraction and chitosan production from *Hermetia illucens* prepupae reared with different substrates. Data represent the mean ± SE (n = 3). For each column significance level at *p* < 0.05. DM, demineralized biomass; DP, deproteinized biomass corresponding to chitin; CHT, unbleached chitosan; RAW, raw dried starting biomass.

	DM/RAW (%)	DP/RAW (%)	CHT/RAW (%)	CHT/CHITIN (%)	Bleached CHT/CHT (%)
Standard	55.09 ± 11.53	6.87 ± 1.06	4.69 ± 0.92	67.22 ± 4.41	13.90 ± 3.19
Fruit	49.37 ± 11.73	5.17 ± 0.29	3.39 ± 0.42	65.24 ± 5.09	13.05 ± 0.02
Meat	35.02 ± 10.40	4.84 ± 0.25	3.30 ± 0.28	68.05 ± 2.19	12.64 ± 2.40
Vegetable	41.00 ± 5.47	4.55 ± 0.93	2.97 ± 0.67	64.64 ± 2.31	19.31 ± 8.20
Mixed	25.84 ± 4.28	3.69 ± 0.33	2.40 ± 0.32	64.91 ± 5.71	18.50 ± 6.81
*p* value	0.2577	0.0700	0.1476	0.9672	0.8124

## Data Availability

Data is contained within the article.
